# A sensitivity analysis of MHD nanofluid flow across an exponentially stretched surface with non-uniform heat flux by response surface methodology

**DOI:** 10.1038/s41598-022-22970-y

**Published:** 2022-11-02

**Authors:** Shahid Hussain, Kianat Rasheed, Aamir Ali, Narcisa Vrinceanu, Ahmed Alshehri, Zahir Shah

**Affiliations:** 1 Department of Mathematics, COMSATS University Islamabad, Attock Campus, Kamra Road, Attock, 43600 Pakistan; 2 Present Address: Department of Industrial Machines and Equipments, Faculty of Engineering, “Lucian Blaga” University of Sibiu, 10 Victoriei Boulevard, 550024 Sibiu, Romania; 3 Department of Mathematics College of Science and Arts, Rabigh King Abdul-Aziz University, Rabigh, 21911 Saudi Arabia; 4 Department of Mathematical Sciences, University of Lakki Marwat, Lakki Marwat, 28420 Khyber Pakhtunkhwa Pakistan

**Keywords:** Engineering, Mathematics and computing

## Abstract

The current study investigates the MHD flow of nanofluid across an elongating surface while taking into account non-uniform heat flux. For this, we have considered the flow of a boundary layer over a stretched sheet containing (water-based) Al_2_O_3_ nanoparticles. The convective boundary conditions for temperature have been invoked. The flow created by a surface that is exponentially expanding in the presence of a magnetic field and a non-uniform heat flux has been mathematically formulated by using laws of conservation. Transformed non-dimensional systems of governing equations have been analyzed numerically by using Adam’s Bashforth predictor corrector approach. The effects of emerging parameters on the fluid velocity and temperature profiles have been further described by plotting graphs. An experimental design and a sensitivity analysis based on Response Surface Methodology (RSM) are used to examine the effects of various physical factors and the dependence of the response factors of interest on the change of the input parameter. To establish the model dependencies of the output response variables, which include the skin friction coefficient and the local Nusselt number, on the independent input parameters, which include the magnetic field parameter, the nanoparticle volume fraction, and the heat transfer Biot number, RSM is used. On the basis of statistical measures such as $Q - Q$ residual plots, adjusted and hypothesis testing using *p* values, it is observed that both of our models for Skin Friction Coefficient (SFC) and the Local Nusselt Number (LNN) are best fitted. Further, it is concluded that the sensitivity of the SFC, as well as the LNN through heat transfer Biot number, is greater than that of nanoparticle volume fraction and magnetic field parameter. The SFC is sensitive to all combinations of the input parameters. At high levels of heat transfer Biot number, the LNN displays negative sensitivity via magnetic field parameters.

## Introduction

Research in the field of fluid mechanics is of more practical importance than research in several other fields because the most commonly encountered substances in human life are fluids like air, water, plasma, mud, colloidal suspensions, blood, etc. The industrial use of these fluids is not possible without complete knowledge of their transport properties. Flows over a stretching surface are encountered in several industrial processes like extrusion of polymer sheets, coating, and colouring of continuously moving metal sheets, drawing of copper wires, thin film coating on photographic films, polyvinyl chloride and plastic sheet extrusion, etc. The study of flows caused by stretching surfaces is a hot topic in current research because of its daily life applications. Sakiadis^1^ was the first to explore the boundary layer flow on a continuously moving surface with a viscous fluid. For 2D flow constrained by a linearly stretching sheet, Crane^2^ obtains a congested type solution. This ground-breaking research for stretching the surface of two-dimensional flows has been examined in a variety of ways. Andersson^3^ has studied the slip effects on a stretching surface. Ariel^4^ extends the work of Crane^2^ to the 3D case. The heat transfer study for electrically conducting fluid of second grade across a stretched surface was given by Liu^5^. Ishak^6^ considers the radiation effects while presenting the MHD flow of the boundary layer owing to an exponentially extending surface. Bachok et al.^7^ investigated the heat transfer consequences of a stagnation point flow across an exponentially extending surface. Mukhopadhyay^8^ employed suction and injection to explore the effects of slip and thermal radiation on boundary layer flow across an exponentially stretched surface using suction and injection. Awais et al.^9^ used computational methods to explore 3D flow across an exponentially elongating surface. Ali et al.^10^ extend their work on three-dimensional flow for non-Newtonian fluids by including the Maxwell nanofluid. Waini et al.^11^ extend the mixed convection flow phenomena for hybrid nanofluid over a exponentially stretching/shrinking surface. Stefan blowing effects for second-grade fluid flow across a curved stretching surface are studied computationally by Gowda et al.^12^. Gowda et al.^13^ also presented the magnetic dipole involvement in ferromagnetic nanofluid flow across a stretched surface. Prasannakumara^14^ discusses the heat transfer phenomenon of the flow of Maxwell nanofluid across a stretching surface and presents the numerical simulations by considering the magnetic dipole effects.

Choi^15^ coined the term “nanofluid” in a report given at the ASME Winter annual meeting. The nanofluids are a novel type of heat transfer fluid that contains both nanoparticles and fluid, i.e., it is a fluid mixture containing a micrometer-sized solid particle that can be used as a heat transfer fluid since the thermal conductivity of these fluids is higher than water. Some recent inventions describe how microparticles can be used in heat transfer applications. The dissipation of nano-size fusible particles in traditional heat transfer fluids is known as nanofluids. The disadvantage of utilizing micro-sized particles (up to 10-6 m) is that waste forms along the flow channel, causing path attrition. This form of fluid has piqued the curiosity of researchers investigating fluids from all around the world. It's found in a wide range of modern technological applications that help people live better lives. Nanofluids are also used in medical applications, such as the treatment of cancerous tumours with gold nanoparticles and the creation of miniature explosives to destroy tumors. Later, Buongiorno^16^ investigated convective heat transmission in nanofluids and discovered that, in the absence of turbulent processes, Brownian diffusion and thermophoresis play a significant role. Nadeem and Lee^17^ investigated the boundary layer flow on an exponentially elongating surface while accounting for the nanofluid. Mustafa et al.^18^ describe boundary layer flow on the exponentially stretched surface using convective boundary conditions. Bhattacharyya and Layek^19^ demonstrated the nanofluid phenomenon across a porous stretched surface. Heat and mass fluxes for nanofluids across a stretched surface are presented by Ghosh and Mukhopadhyay^20^. Sulaiman et al.^21^ discussed the 3D flow of nanofluid-containing micro-organisms. The heat transfer analysis of nanofluid across an exponentially diminishing surface with heat and mass fluxes was described by Ghosh and Mukhopadhyay^22^. A nanofluid phenomenon over an exponentially stretching surface has been presented by Ali et al.^23^ by considering the effects of non-uniform heat flux and convective walls. Oldroyd-B fluid flows across an exponentially extending surface for 3D. Ali et al.^24^ presented 3D flow across an exponentially extending surface for Oldroyd-B fluid. Recently, the entropy generation analysis of peristaltic flow for nanofluid has been investigated by Ali et al.^25^. By utilizing the Koo-Kleinstreuer and Li (KKL) correlation, Gowda et al.^26^ examined the modified Fourier heat flux model for the nanofluid flow on a curved stretched surface. In order to study the effects of a porous medium on the nanofluid flow on a rotating disk, Kumar et al.^27^ took into account Darcy-Forchheimer phenomena. Gowda et al.^28^ present thermophoretic particle statements in the time-dependent flow of hybrid nanofluid across a rotating disk. A discussion on the entropy generation phenomena for the Marangoni flow by considering the nanoparticles of aluminium oxide and copper has been presented by Li et al.^29^. Yusuf et al.^30^ investigated the phenomenon of gyrotactic microorganisms for the flow of non-Newtonian Williamson nanofluid across an inclined surface. Mahanthesh^31,32^ studied the aggregation kinematics of nanoparticles as well as the flow and heat transfer analysis of nanomaterials with quadratic radiative heat flux. Sheikholeslami and Ebrahimpour^33^ investigated the Linear Fresnel Reflector (LFR) solar system by employing multi-way twisted tape using Al_2_O_3_-water nanofluid. They use a finite volume technique for simulations. Sheikholeslami et al.^34^ presented the numerical simulations for the thermal analysis of PTC with a new wavy absorber tube within a solar system. They consider a two-phase model of nanofluid with a mixture of nanoparticles of oil and CuO. They conclude that by increasing the Reynolds number from 5000 to 20,000, friction factor is reduced by 28.96% with an improvement of 180.13% in heat transfer coefficient. Sheikholeslami^35^ also presented the air conditioning unit that involves porous media, which consists of five-lobed cylinders containing paraffin and nano-sized particles of ZnO. Gowda et al.^36^ inspected the slip effects on the flow of Casson-Maxwell nanofluid inside stretchable disks. Sheikholeslami^37–39^ also discusses the numerical analysis of a solar system equipped with a novel turbulator and analyze the melting process of paraffin using a honeycomb-shaped heat storage device. Additionally, for the purpose of melting, they statistically analyze the solar energy storage within a double pipe.

The study of the movements of electrically conducting fluids is known as magnetohydrodynamic (MHD). Solitons, liquid crystals, seawater, and electrodes are just a few examples of this type of fluid. The term magnetohydrodynamic is made up of three words: magneto (which refers to a magnetic field); hydro (which refers to a liquid); and dynamic (which refers to motion). According to Faraday's law of electromagnetic induction, when an electric field and a magnetic field move relative to one another, a potential is formed in the conductor, causing current to flow between the endpoints. This law is used to generate MHD electricity. Magnetic fields may cause currents to flow through a flowing electrically conductive fluid, polarizing the fluid and changing the magnetic field in the process. Alfven^40^ used the term magnetohydrodynamics (MHD) to describe such a fluid. Many studies^41–46^ have been conducted to better understand the transport mechanisms and novel applications of MHD flows, which have proven to be advantageous in a variety of industrial processes such as polymer extrusion, hot rolling, and stretching of plastic films, wire drawing, metal extrusion, glass-fiber, and metal spinning. Benos et al.^47^ investigated the MHD natural convection flow of a CNT-water nanofluid theoretically by incorporating the Hamilton-Crosser model.

Researchers have recently concentrated on using statistical tools to analyze the effects of various physical characteristics. To address the issues raised by earlier reviewers, it was decided to do a statistical analysis of the findings of the accessible publications in the literature. The statistical analysis would allow for the representation of observations on complete charts (histograms and scatter diagrams), allowing for a more solid and mathematically reliable extraction of findings. All of the observations accessible in the literature are given the same amount of weight in this literature evaluation. In industrial and applied research, experimental design is crucial. When a combination of input parameter values is applied to an experimental unit, one or more responses are measured over the experimental unit. Box and Wilson’s Response Surface Methodology (RSM) is a feasible approach in experimental design for proper observation of the mechanism and computing the values of input parameters that optimize the response. Different studies using RSM have been done; a few of them can be seen in^48–52^ and in their references. Researchers can utilize RSM to help them create a list of experimental designs which can be used to predict response. It might be beneficial to alter the theoretical constraints to examine a certain model term or interaction. Furthermore, it may recommend the best amount or value of input parameters to maximize the answer. In related research, RSM is commonly used to find the optimal parameter level. Sensitivity analysis is a procedure that involves changing one or more variables in a problem to assess how such changes may affect a result or quantity of interest. Such a procedure has likely been used in all branches of science for a very long time. The impact of a problem restriction on the optimality of a cost or benefit function via shadow pricing, for instance, or the role and function of a model parameter in producing a model output, are a few examples. Because the sensitivity of the problem is only evaluated around a nominal point in the problem space, these analyses are frequently referred to as “Local Sensitivity Analysis (LSA)”. LSA is easy to use, intuitive, and suitable for extremely certain situations. Although it has been criticized for simply offering a confined picture of the issue space, it has still been utilized frequently and much more widely, particularly when employed in the context of examining parameter relevance in mathematical modelling. Additionally, sensitivity analysis is frequently used in conjunction with an experimental approach like RSM to assess how much the response depends on the input parameters^53–58^. The sensitivity analysis of nonlinear convective heat transfer in hybrid nanomaterial inside two concentric cylinders with non-uniform heat sources is covered by Thriveni and Mahanthesh^59^. A sensitivity analysis is carried out by Mackolil and Mahanthesh^60^ to optimize heat transmission in the flow of thermal Marangoni convective hybrid nanomaterial. In a hybrid nanofluid with a thermal radiation effect, melting heat transfer is demonstrated by Basir et al.^61^ using a stability study. Using the modified Buongiorno model, Mahanthesh et al.^62^ carried out a sensitivity analysis on the flow of nanofluid. A sensitivity analysis on the impact of the nonlinear Boussinesq approximation with non-uniform heat source/sink on the flow of nanofluid under convective heat conditions was provided by Mahanthesh et al.^63^. The sensitivity analysis of the flow of nanofluid in the presence of thermal Marangoni convection and inclined magnetic field effects was studied by Mackolil and Mahanthesh^64^.

The main goal of this study is to examine the influence of different physical factors and the dependence of the response parameters of interest on the change of input parameters through experimental design and sensitivity analysis based on response surface methodology. Experimental design is important in both applied and industrial research. In a designed experiment, when a variety of input parameter levels are applied to the testing unit, one or more responses are over the experimental units. Motivated by all these applications and by utilizing the knowledge of the above mentioned literature, the objective of this study is to apply an experimental design and a sensitivity analysis to the problem of MHD flow of nanofluid across an exponentially stretching surface in the presence of non-uniform heat flux. A nanofluid is a novel type of heat transfer fluid that comprises nanoparticles in the base fluid. It has many industrial applications, such as transportation, power generation, pharmaceutical processes, micro-manufacturing, etc. It can be used for thermal therapy for cancer treatment. Because of its vast variety of industrial uses, the flow of nanofluid across a stretched surface has become highly popular among contemporary researchers. Plastic sheet extrusion, hot rolling, wire drawing, paper manufacture, plastic film drawing, fiber glass, and cooling of a metallic plate are examples of such mechanisms. Scientists are still looking for the best ways to use these nanofluids and are attempting to minimize any bad features that may arise when they are used in a project or system. This study also looked at the impact of non-uniform heat flux on the flow of nanofluid through an elongating surface. In the current analysis, mathematical formulation was performed using conservation laws and appropriate transformations to simplify our set of nonlinear coupled partial differential equations into a set of coupled nonlinear ordinary differential equations. The numerical solution of a transformed set of non-dimensional governing equations was obtained using Mathematica’s NDSolve command. The effects of emerging parameters have been further explained by plotting graphs and analyzing the results using physical descriptions. Further, a data set is developed through a numerical technique because we have used an experimental design-based approach to determine the significance of specific input parameters that may improve response. The experimental design-based analysis is the main achievement of this research for the flow of nanofluid over an exponentially stretched surface.

## Mathematical formulation

**Figure 1:**
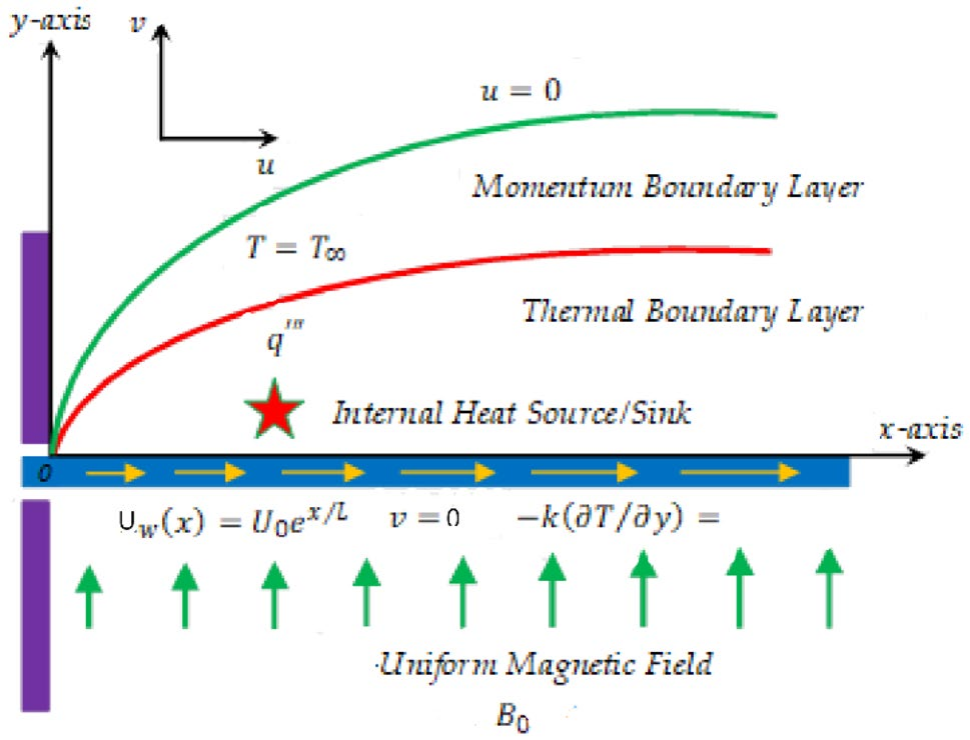
Geometry of the problem.

To understand the properties of the thermal transfer mechanism along an exponentially extending surface, we have considered a steady, incompressible, 2D flow of viscous base nanofluid across an exponentially stretched surface. A uniform magnetic field has been applied normal to the surface to examine the effects of MHD. The effects of non-uniform heat flux and the convective boundary conditions are also considered. A cartesian coordinate system has been used in which the *x*—axis is taken parallel to the stretched surface and the *y*—axis is normal to it. The plate has been stretched in the $x -$ direction with velocity $U_{w} \left( x \right) = U_{0} \exp \left( \frac{x}{l} \right)$, in which $U_{0} \;{\text{ and}}\; \, l$ is the reference velocity and characteristic length. Figure 1 depicts the geometry of the model under consideration.

The following are the mass, momentum, and energy equations after using boundary layer approximations for nanofluid flow past a stretching surface with a magnetic field and non-uniform heat flux effects^23^:

1 $$\nabla \cdot {\mathbf{V}} = 0,$$

2 $$\rho_{nf} \frac{{d{\mathbf{V}}}}{dt} = \nabla \cdot {\mathbf{T}} + {\mathbf{J}} \times {\mathbf{B}},$$

3 $$\left( {\rho c_{p} } \right)_{nf} \frac{dT}{{dt}} = \kappa_{nf} \nabla^{2} T + q^{\prime\prime\prime}.$$

where ${\mathbf{V}} = \left[ {u\left( {x,y} \right),v\left( {x,y} \right),0} \right]$ is the velocity field, $\frac{d}{dt}$ is the material time derivative, ${\mathbf{J}} \times {\mathbf{B}}$ is the Lorentz force vector calculated from Ohm’s law, $\rho_{nf}$ is the density of nanofluid $\left( {\rho c_{p} } \right)_{nf}$ is the heat capacity of nanofluid, $T$ is the fluid temperature, and $\kappa_{nf}$ is the thermal conductivity of nanofluid, and $q^{\prime\prime\prime}$ is a non-uniform heat source/sink parameter defined as follows ^23^:

4 $$q^{\prime \prime \prime } = \frac{{\kappa_{nf} U_{w} \left( x \right)}}{{x\upsilon_{nf} }}\left[ {A^{*} \left( {T_{w} - T_{\infty } } \right)f^{\prime } + \left( {T - T_{\infty } } \right)B^{*} } \right],$$

where $A^{*} {\text{ and }}B^{*}$ are, respectively, the space-dependent and time-dependent heat source and sink coefficients; $T_{w}$ the wall temperature; and $T_{\infty }$ the ambient (far away from the surface) temperature. Under the given assumptions, conservations laws of mass, momentum and energy Eqs. (1)–(3), in the presence of non-uniform heat generation/absorption (4) and using boundary layer approximations takes the following form^23^:

5 $$\frac{\partial u}{{\partial x}} + \frac{\partial v}{{\partial y}} = 0$$

6 $$u\frac{\partial u}{{\partial x}} + v\frac{\partial u}{{\partial y}} = \frac{{\mu_{nf} }}{{\rho_{nf} }}\frac{{\partial^{2} u}}{{\partial y^{2} }} - \frac{{\sigma_{nf} }}{{\rho_{nf} }}B_{0}^{2} u,$$

7 $$u\frac{\partial T}{{\partial x}} + v\frac{\partial T}{{\partial y}} = \frac{{\kappa_{nf} }}{{\left( {\rho c_{p} } \right)_{nf} }}\frac{{\partial^{2} T}}{{\partial y^{2} }} + \frac{{\kappa_{nf} U_{w} \left( x \right)}}{{x\upsilon_{nf} \left( {\rho c_{p} } \right)_{nf} }}\left[ {A^{*} \left( {T_{w} - T_{\infty } } \right)f^{\prime} + \left( {T - T_{\infty } } \right)B^{*} } \right].$$

The following are the boundary conditions for the considered problem^23^:

8 $$\begin{array}{ll} {u = U_{w} \left( x \right) = U_{o} \exp \left( \frac{x}{l} \right),\quad v = 0,\quad - \kappa_{f} \frac{\partial T}{{\partial y}} = h_{f} \left( {T_{w} - T_{\infty } } \right),} & {{\text{at}}\;y = 0,} \\ {u \to 0,\;T \to T_{\infty } } & {{\text{as}}\;y \to \infty .} \\ \end{array}$$

In the preceding equations, $$\left( {u,v} \right)$$ are the velocity components which are taken along and normal to the stretched surface, $$T$$ the fluid temperature, $$B_{0}$$ the magnetic field strength, $$\mu_{nf} ,$$$$\rho_{nf} ,$$$$\sigma_{nf} ,$$$$\kappa_{nf} ,$$$${\text{and }}\left( {\rho c_{p} } \right)_{nf}$$ are the viscosity coefficient, the density, the electrical conductivity, the thermal conductivity, and the heat capacity of nanofluid, respectively.

Let us define the following non-dimensional variables and transformations to get the non-dimensional form of Eqs. (1)–(3).

9 $$\begin{aligned} & u = U_{0} \exp \left( \frac{x}{l} \right)f^{\prime } \left( \eta \right),\quad v = - \sqrt {\frac{{\upsilon_{f} U_{0} }}{2l}} \exp \left( \frac{x}{l} \right)\left[ {f\left( \eta \right) + \eta f^{\prime } \left( \eta \right)} \right], \\ & \theta \left( \eta \right) = \frac{{T - T_{\infty } }}{{T_{w} - T_{\infty } }},\quad \eta = y\sqrt {\frac{{U_{0} }}{{2\upsilon_{f} l}}} \exp \left( \frac{x}{2l} \right). \\ \end{aligned}$$

The continuity Eq. (5) is identically satisfied by inserting Eq. (9) into the Eqs. (5)–(7), whereas Eqs. (6) and (7) take the following non-dimensional form:

10 $$\frac{{A_{0} }}{{A_{1} }}f^{\prime \prime \prime } + ff^{\prime \prime } - 2f^{\prime 2} - \frac{{A_{2} }}{{A_{1} }}Mf^{\prime } = 0,$$

11 $$\theta^{\prime \prime } + \Pr \frac{{A_{3} }}{{A_{4} }}f\theta^{\prime } + \frac{{A_{1} }}{{A_{0} }}\left( {Af^{\prime } + B\theta } \right) = 0,$$

Similarly, by using (9) in place of boundary conditions (8), we get the following non-dimensional form of boundary conditions:

12 $$\begin{aligned} & f\left( \eta \right) = 0,\; \, f^{\prime } \left( \eta \right) = 1, \, \;\theta^{\prime } \left( \eta \right) = - \gamma \left( {1 - \theta \left( \eta \right)} \right)\quad {\text{at }}\quad \eta = 0, \\ \, & f^{\prime } \left( \eta \right) \to 0,\;\theta \left( \eta \right) \to 0\;\;{\text{as}}\;\;\eta \to \infty , \\ \end{aligned}$$

In the non-dimensional Eqs. (10)–(11), the constants appearing due to nanofluid particles are:

13 $$A_{0} = \frac{{\mu_{nf} }}{{\mu_{f} }},\,\,A_{1} = \frac{{\rho_{nf} }}{{\rho_{f} }},\,A_{2} = \frac{{\sigma_{nf} }}{{\sigma_{f} }},\,A_{3} = \frac{{\left( {\rho c_{p} } \right)_{nf} }}{{\left( {\rho c_{p} } \right)_{f} }},\,A_{4} = \frac{{\kappa_{nf} }}{{\kappa_{f} }}.$$

where the expressions for the thermal properties of nanofluid are defined in Table 1. Furthermore, the Hartmann and Prandtl numbers are defined as $M = \left( {\frac{{\sigma_{f} B_{0}^{2} U_{0} }}{{\rho_{f} }}} \right)$ and $\Pr = \frac{{\mu_{f} \left( {\rho c_{p} } \right)_{f} }}{{\rho_{f} \kappa_{f} }}$. The physical quantities of interest, namely SFC and LNN, may be stated mathematically as follows:

14 $$\begin{array}{*{20}c} {Cf = \frac{{\tau_{w} }}{{\rho_{f} U_{w}^{2} }}{,}} & {\tau_{w} = \mu_{nf} \left( {\frac{ \, \partial u}{{\partial y}}} \right)_{y = 0} } \\ {Nu = \frac{{xq_{w} }}{{\kappa_{f} \left( {T_{w} - T_{\infty } } \right)}}{ ,}} & {q_{w} = - \kappa_{nf} \left( {\frac{ \, \partial T}{{\partial y}}} \right)_{y = 0} } \\ \end{array}$$

**Table 1:** Mathematical expressions for nanofluid thermo-physical properties.

Properties	Nanofluid
Density	$\rho_{nf} = \left( {1 - \phi } \right)\rho_{f} + \phi \rho_{s}$
Viscosity	$\mu_{nf} = \frac{{\mu_{f} }}{{\left( {1 - \phi } \right)^{2.5} }}$
Heat capacity	$\left( {\rho c_{p} } \right)_{nf} = \left( {1 - \phi } \right)\left( {\rho c_{p} } \right)_{f} + \phi \left( {\rho c_{p} } \right)_{s} ,$
Thermal conductivity	$\frac{{\kappa_{nf} }}{{\kappa_{f} }} = \frac{{\kappa_{s} + \left( {n - 1} \right)\kappa_{f} - \left( {n - 1} \right)\phi \left( {\kappa_{f} - \kappa_{s} } \right)}}{{\kappa_{s} + \left( {n - 1} \right)\kappa_{f} + \phi \left( {\kappa_{f} - \kappa_{s} } \right)}}$
Electric conductivity	$\,\frac{{\sigma_{nf} }}{{\sigma_{f} }} = \frac{{\sigma_{s} + 2\sigma_{f} - 2\phi \left( {\sigma_{f} - \sigma_{s} } \right)}}{{\sigma_{s} + 2\sigma_{f} + \phi \left( {\sigma_{f} - \sigma_{s} } \right)}}.$

The non-dimensional form of SFC and LNN after using Eq. (9) is:

15 $$Cf\sqrt {{\text{Re}}_{x} } = f^{\prime \prime } \left( 0 \right),\,\,Nu/\sqrt {{\text{Re}}_{x} } = \theta^{\prime } \left( 0 \right).$$

Here ${\text{Re}}_{x} = \frac{{U_{w} x}}{{\nu_{f} }},$ is local Reynolds number.

## Thermo-physical properties

For this problem, we used nanoparticles of aluminium oxide (Al_2_O_3_) and water (H_2_O) as the base fluid. Table 1 depicts the fundamental thermo-physical characteristics of nanofluids, where the subscripts $nf,\,f,\,s$ signify nanofluid, base fluid, and nanoparticles, respectively. Table 2 shows the numerical values for the thermo-physical properties of nanoparticles and base fluid utilized to generate the computational results.

**Table 2:** Numerical values of the thermo-physical properties of nanoparticles and base fluid.

Physical properties	Al_2_O_3_	H_2_O
$\rho \left( {kgm^{ - 3} } \right)$	3970	997.1
$c_{p} \left( {J{\text{ kg}}^{{ - {1}}} {\rm K}^{ - 1} } \right)$	765	4179
$\kappa \,\left( {W\;{\text{ m}}^{{ - {1}}} \, \;{\rm K}^{{ - {1}}} } \right)$	40	0.614
$\sigma \left( {\Omega^{ - 1} m^{ - 1} } \right)$	$35 \times 10^{6}$	$5.5 \times 10^{ - 6}$

## Results and discussion

**Figure 2:**
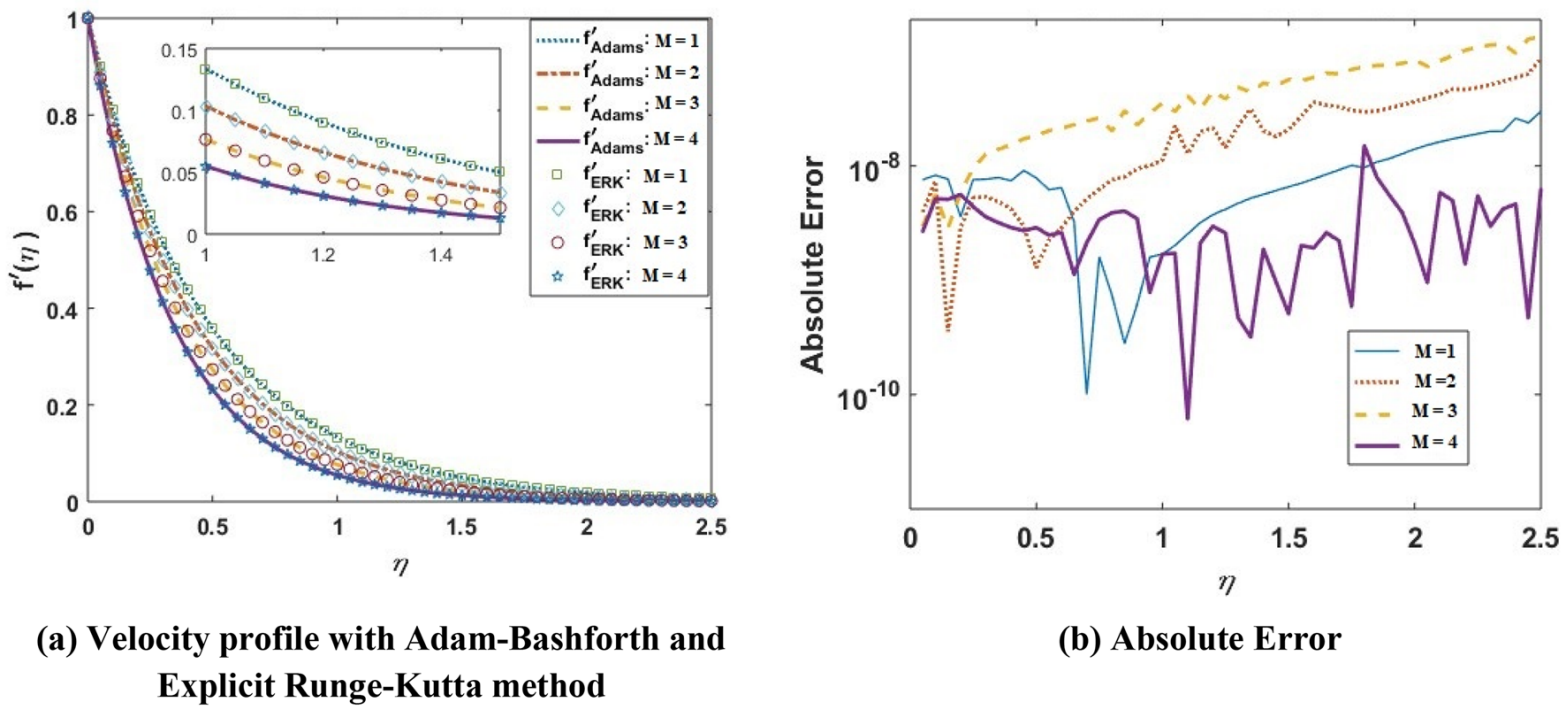
Velocity profile and absolute error for varying values of $M$.

This section shows how various quantities affect the flow profiles generated by a numerical technique. The Adam’s-Bashforth predictor corrector method, being a linear multistep technique, is employed for this purpose. Multistep techniques try to enhance effectiveness by retaining and using previous phase information instead of deleting it, and hence make use of such a large number of earlier points and derivative values. It works in two phases: firstly, we have used the Adam’s-Bashforth technique to generate an approximation of the desired values as a predictor. Then, we use Adam’s Molten as a corrector step that rectifies an initial estimate. For the validation of the results obtained from the Adam-Bashforth predictor–corrector technique, we have compared our results with those obtained from the explicit Runge–Kutta method. In Fig. 2a, we plot the velocity profile obtained from both the Adam-Bashforth method and the explicit Runge–Kutta method for varying values of the magnetic field parameter $M$. In Fig. 2b, we present the absolute error for different values of $M$. It is observed that the absolute error between the results obtained from the Adam-Bashforth method and the explicit Runge–Kutta method is around $10^{ - 6} - 10^{ - 10}$, which is in a negligible vicinity, and hence validates our results.

**Figure 3:**
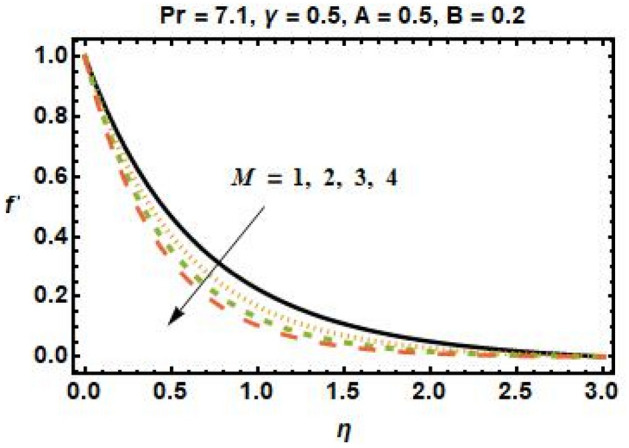
Effects of magnetic field parameter on the velocity field.

**Figure 4:**
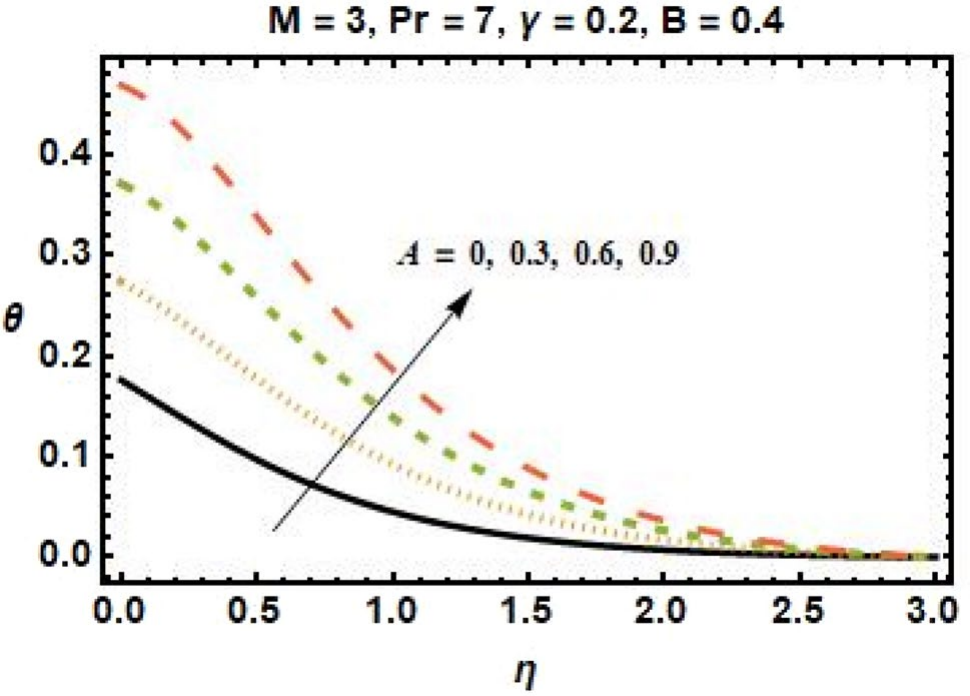
Effects of space-dependent parameter on the temperature profile for $A > 0$.

**Figure 5:**
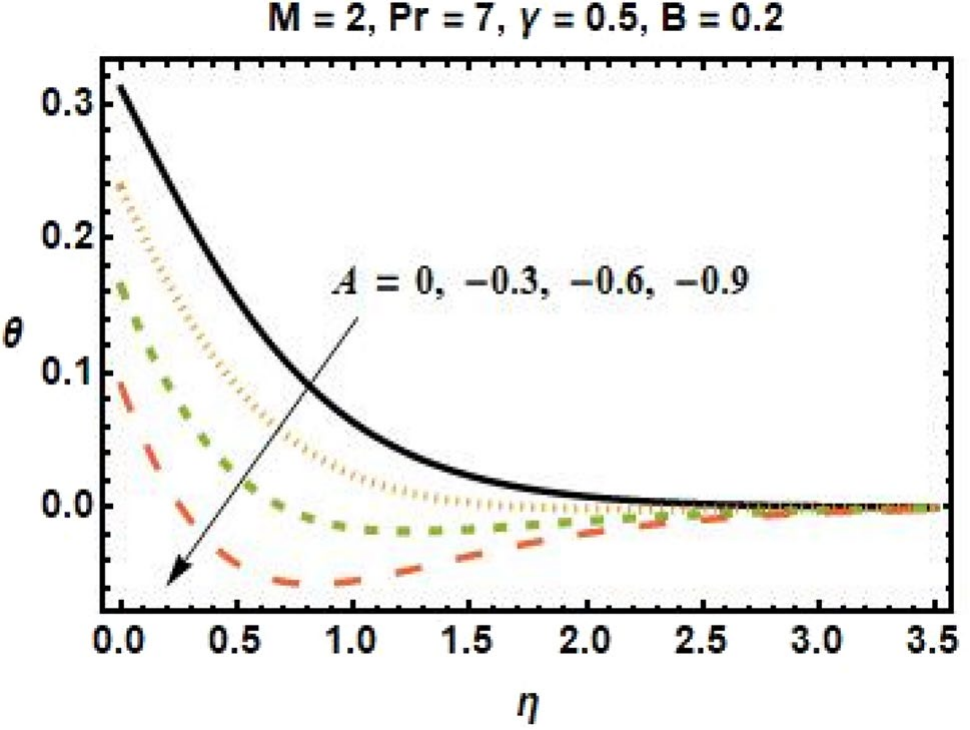
Effects of space-dependent parameter on the temperature profile for $A < 0$.

**Figure 6:**
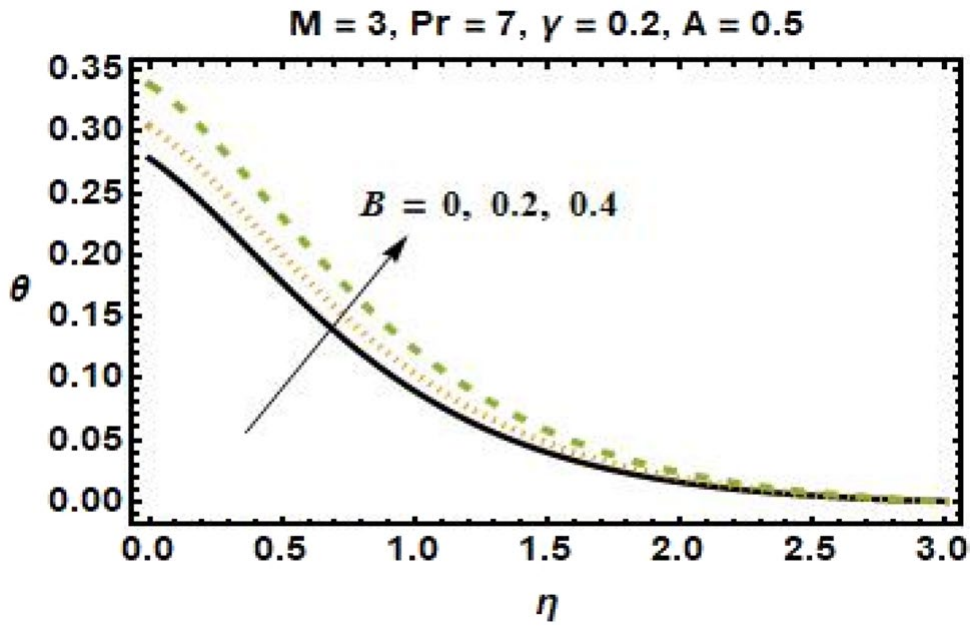
Effects of time-dependent parameter on the temperature profile for $B > 0$.

**Figure 7:**
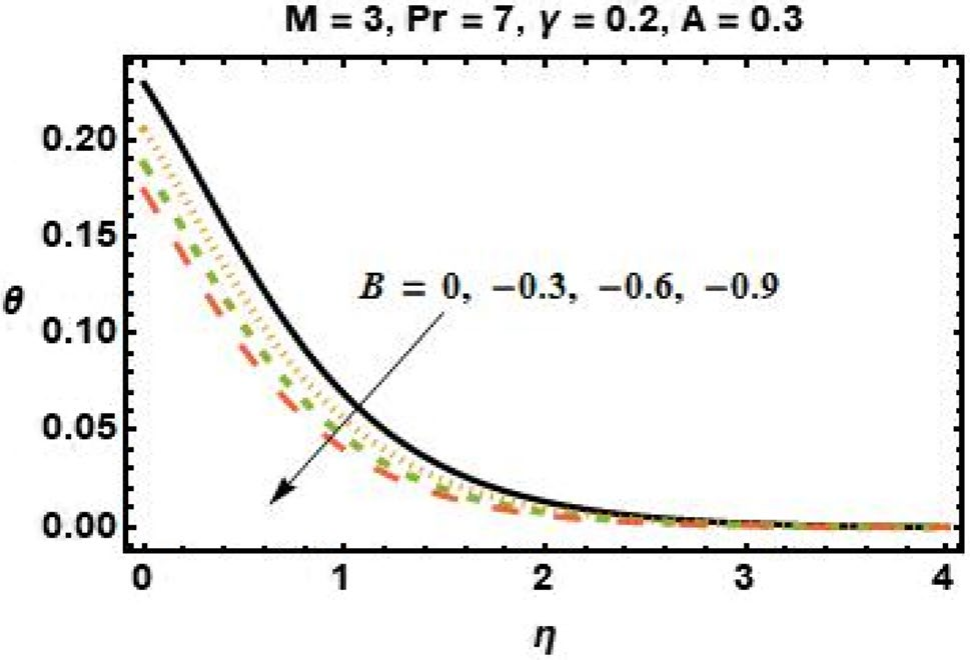
Effects of time-dependent parameter on the temperature profile for $B < 0$.

**Figure 8:**
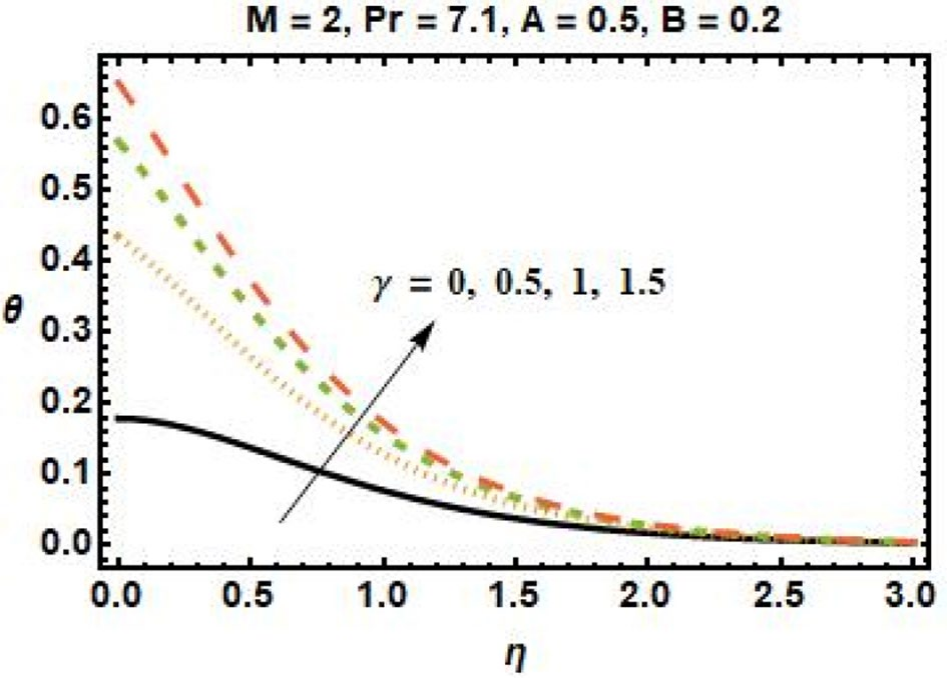
Temperature profiles for different values of the thermal relaxation parameter.

**Figure 9:**
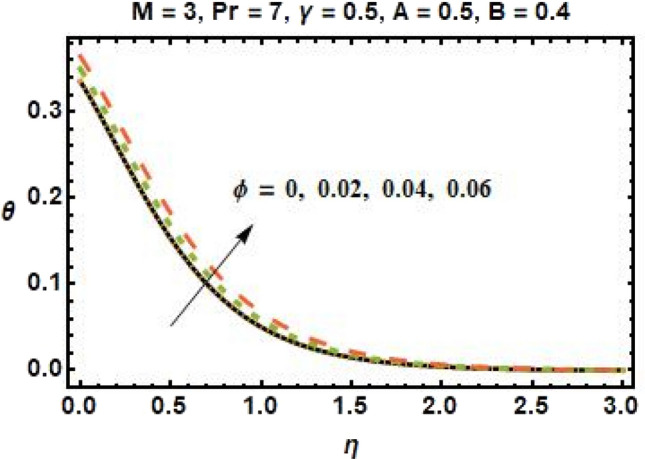
Temperature profiles for different values of volume fraction of nanoparticles.

Further, a data set is developed through a numerical technique because we have used an experimental design-based approach to determine the significance of specific input parameters that may improve response. Physical changes in velocity and temperature fields are depicted in Figs. 3, 4, 5, 6, 7, 8, and 9 as a function of parameters such as Hartmann number ($M$), nanoparticle volume fraction ($\phi$), heat source and sink parameters ($A{\text{ and }}B$), and thermal relaxation parameter ($\gamma$). Figure 3 depicts the dependence of velocity on the magnetic field parameter. The velocity field is seen to decrease as $M$ increases. The Lorentz force operates as a decelerator, reducing the speed of the fluid and the momentum boundary layer thickness. As a result, larger $M$ values intensify the resistive force, which resists the magnetic forces with dominant retarding effects and reduces fluid velocity.

Figures 4, 5, 6, and 7 show the impact of different values of space-dependent and time-dependent parameters $A{\text{ and }}B$ on fluid temperature for both cases of heat generation and internal heat absorption. An increase in the temperature of the fluid is noted in the case of $A > 0$ but decreases in the case of $A < 0$. The momentum boundary layer decreases $A > 0$, which tends to slow down the velocity in the boundary layer, causing the temperature profile to increase, implying that when $A$ improved. The existence of $A$ physically reduces the quantity of heat available to the system, resulting in a degraded transportation mechanism. In the presence of a magnetic field $M$, the thermal boundary layer expands for $A < 0$. The temperature of the fluid tends to rise due to the existence of a heat source in the boundary layer that generates energy. Figures 6 and 7 depict the effects of time-dependent heat source and sink parameters on fluid temperature. The temperature of the fluid rises $B > 0$ and falls $B < 0$. Heat generation (a non-uniform heat source parameter greater than zero) boosts fluid temperature by adding extra heat into the system and thickens the thermal boundary layer. The existence of a heat source in the interior causes the flow field to transmit extra heat, which results in the thermal boundary layer thickness. This $B < 0$ works as a heat absorber, causing the fluid to lose heat as it cools, resulting in a fall in fluid temperature.

Improved heat conductivity is one of the most essential features. Thermal conductivity enhancements can result in slight efficiency gains due to more efficient fluid heat transfer. Increasing the quantity of nanoparticles added to the base fluid improves the heat transmission properties of the material, resulting in a higher temperature profile. The impact of the heat transfer Biot number on the dimensionless temperature field is seen in Fig. 8. The increase in $\gamma$ implies that the heat transfer coefficient is increasing as well, and so does the temperature. The flow in the boundary layer moves as a function of the velocity of the stretched sheet. As a result, increasing the Biot number reduces the impact of sheet motion on the boundary layer, slowing the flow. Figure 9 illustrates the influence of $\phi$ on fluid temperature. According to research, the temperature rises as we increase the quantity of nanoparticles in the base fluid. This is because increasing the number of nanoparticles improves the thermal properties of fluids. Furthermore, adding nanoparticles to the base fluid boosts the heat transmission capabilities of the material, resulting in a higher temperature profile.

## Experimental design

In mathematical modelling, an experimental scheme is important because numerical computational simulations have a better chance of being interpreted. A data set is developed through computer programming and applied to real-world scenarios. Response Surface Methodology (RSM) is an experimental design-based approach to determine which input parameter is significant and which particular level of an input parameter may improve response, since numerous input parameters might affect response. The RSM is used to establish the model dependencies of the output response variables, which include the SFC ($Cf\sqrt {{\text{Re}}_{x} }$) and the LNN ($Nu/\sqrt {{\text{Re}}_{x} }$), on the independent input parameters, which include different parameters like the magnetic field ($M$), the nanoparticle volume fraction ($\phi$), and the heat transfer Biot number ($\gamma$). The sensitivity analysis is also performed on one of the numerous model coefficients and parameters, but only for the relevant response variables and parameters indicated above. The typical nonlinear polynomial model is used to study and assess the correlation between the specified response variable model parameters, which is presented as:

16 $$\hat{R} = r_{0} + r_{1} A + r_{2} B + r_{3} C + r_{11} A^{2} + r_{22} B^{2} + r_{33} C^{2} + r_{12} AB + r_{23} BC + r_{13} AC$$

There is an intercept ($r_{0}$), three linear terms ($r_{1} ,r_{2} ,r_{3}$), three square terms ($r_{11} ,r_{22} ,r_{33}$), and three interaction terms ($r_{12} ,r_{23} ,r_{13}$) in this response surface Eq. (16). The SFC and the LNN are denoted by the response ($\hat{R}$). For two responses, two response surface equations are investigated. The magnetic field parameter ($M$), nanoparticle volume fraction ($\phi$), and heat transfer Biot number ($\gamma$) are represented by the coded symbols *A, B,* and *C,* respectively. Three levels are chosen for each of these characteristics: low, medium, and high, denoted by (− 1, 0, 1). Table 3 shows the input values as well as the nomenclature for them. A response surface element has been introduced to determine the experimental outcomes. It is determined that 20 runs and 19 degrees of freedom are appropriate for three levels of specified parameters. The number of runs for the experimental investigation is calculated by the formula ${2}^{F} + 2F + C,$ where $F = 3$ denotes the number of factors and $C = 6$ denotes the number of centre points. Table 4 displays the outcomes of these runs. Table 5 shows their estimates, as well as *t*-values and *p*-values, to study the relevant input parameters further. This indicates that $\phi$ ($p{\text{ - value}}\, < {7}{\text{.68}} \times {10}^{ - 11}$) and $\gamma$ ($p{\text{ - value}}\, < {3}{\text{.41}} \times {10}^{ - 13}$) are significant terms with effect $Cf\sqrt {{\text{Re}}_{x} }$. Also, $\phi$ ($p{\text{ - value}} < { 5}{\text{.62}} \times {10}^{ - 12}$) and $\gamma$ ($p{\text{ - value}} < {3}{\text{.41}} \times {10}^{ - 13}$) are significant terms that influence $Nu/\sqrt {{\text{Re}}_{x} }$.

**Table 3:** Symbols and levels of experimental parameters.

Parameter	Symbol	Low (−1)	Medium (0)	High (1)
$M$	*A*	0.5	1	1.5
$\phi$	*B*	0.01	0.05	0.09
$\gamma$	*C*	0.5	2	3.5

**Table 4:** Response results for $Cf\sqrt {{\text{Re}}_{x} }$ and $Nu/\sqrt {{\text{Re}}_{x} }$ regarding RSM design.

Run	$\phi$	$M$	$\gamma$	*A*	*B*	*C*	$Cf\sqrt {{\text{Re}}_{x} }$	$Nu/\sqrt {{\text{Re}}_{x} }$
1	0.01	1	3.5	−1	0	1	21.21918	3.80955
2	0.01	1	0.5	−1	0	−1	4.50138	0.37485
3	0.05	1	2	0	0	0	11.294	1.57933
4	0.09	0.5	2	1	−1	0	7.77798	1.45778
5	0.05	1	2	0	0	0	11.294	1.57933
6	0.09	1	3.5	1	0	1	21.56398	3.90887
7	0.05	1	2	0	0	0	11.294	1.57933
8	0.05	0.5	0.5	0	−1	−1	0.6925	0.31139
9	0.05	1.5	0.5	0	1	−1	10.256	0.47191
10	0.09	1	0.5	1	0	−1	4.54378	0.40457
11	0.05	1	2	0	0	0	11.294	1.57933
12	0.05	0.5	3.5	0	−1	1	15.7315	3.64676
13	0.05	1	2	0	0	0	11.294	1.57933
14	0.05	1.5	3.5	0	1	1	28.1615	4.17891
15	0.09	1.5	2	1	1	0	16.09978	1.77215
16	0.01	0.5	2	−1	−1	0	7.62542	1.41299
17	0.05	1	2	0	0	0	11.294	1.57933
18	0.01	1.5	2	−1	1	0	15.86538	1.69592
19	0.01	1	2	0	0	0	11.294	1.57933
20	0.01	1	2	0	0	0	11.294	1.57933

**Table 5:** Evaluations of fitted model terms, along with *t*-value and *p*-value.

Term	Estimate	SE	*t*-value	*p*-value
$$\user2{Cf}\sqrt {{\mathbf{Re}}_{\user2{x}} }$$				
Intercept	11.294	0.21699	52.0491	${ 1}{\text{.66}} \times {10}^{ - 13}$
A $\left( M \right)$	0.09677	0.21699	0.446	0.665119
B $\left( \phi \right)$	4.81941	0.21699	22.2105	${7}{\text{.68}} \times {10}^{ - 10}$
C $\left( \gamma \right)$	8.33531	0.21699	38.4138	${3}{\text{.41}} \times {10}^{ - 12}$
AB $\left( {M\phi } \right)$	0.02046	0.30687	0.0667	0.948156
AC $\left( {\phi \gamma } \right)$	0.0756	0.30687	0.2464	0.810386
BC $\left( {M\gamma } \right)$	0.71662	0.30687	2.3353	0.041672
A^2^ $\left( {M^{2} } \right)$	−0.10258	0.28705	−0.3574	0.728253
B^2^ $\left( {\phi^{2} } \right)$	0.65072	0.28705	2.2669	0.046814
C^2^ $\left( {\gamma^{2} } \right)$	1.76566	0.28705	6.1511	0.000108
$$\user2{Nu}/\sqrt {{\mathbf{Re}}_{\user2{x}} }$$				
Intercept	1.57933	0.00556	283.619	$< \,\,2.2 \times 10^{ - 16}$
A $\left( M \right)$	0.031258	0.00556	5.6133	0.000224
B $\left( \phi \right)$	0.161246	0.00556	28.9569	${ 5}{\text{.62}} \times {10}^{ - 11}$
C $\left( \gamma \right)$	1.747671	0.00556	313.850	$\, < \, 2.2 \times 10^{ - 16}$
AB $\left( {M\phi } \right)$	0.00786	0.00787	0.9981	0.341773
AC $\left( {\phi \gamma } \right)$	0.0174	0.00787	2.2095	0.051603
BC $\left( {M\gamma } \right)$	0.092908	0.00787	11.7977	$3.43 \times 10^{ - 7}$
A^2^ $\left( {M^{2} } \right)$	−0.0112	0.00736	−1.5206	0.159333
B^2^ $\phi^{2}$	0.016581	0.00736	2.2509	0.159333
C^2^ $\left( {\gamma^{2} } \right)$	0.556331	0.00736	75.5227	$4.04 \times 10^{ - 15}$

Table 6 represents the results of the response surface based ANOVA (analysis of variance) performed to assess the effect of independent input parameters on $Cf\sqrt {{\text{Re}}_{x} }$ and $Nu/\sqrt {{\text{Re}}_{x} }$. A level of flexibility, SS (sum of squares), MS (mean of sum of squares), *F*-statistics, and *p*-value are among the outputs. In an ANOVA investigation, the estimation of data variance across average value and the probability validation of model correctness are represented in statistical contexts by *F*-value and *p*-value, respectively. A high *F*-value and a modest *p*-value provide sufficient evidence for the importance of the outcome. As a result, both *F*-value and *p*-value are frequently used to assess the significance of response variables. The components tested, namely $\phi$, $M$, and $\gamma$, are found to have a substantial linear impact ($p - {\text{value}} < 0.0001$) on both responses ($Cf\sqrt {{\text{Re}}_{x} }$ and $Nu/\sqrt {{\text{Re}}_{x} }$). In the same way, the model parameter has a quadratic influence on all the outcomes. Furthermore, the model’s interaction impact is significant for $Cf\sqrt {{\text{Re}}_{x} }$ and $Nu/\sqrt {{\text{Re}}_{x} }$.

**Table 6:** ANOVA analysis for $Cf\sqrt {{\text{Re}}_{x} }$ and $Nu/\sqrt {{\text{Re}}_{x} }$.

Source	DOF	SS	Contribution	MS	*F*-value	*p*-value
$$\user2{Cf}\sqrt {{\mathbf{Re}}_{\user2{x}} }$$						
Linear	3	741.71	96.348	247.236	$6.56 \times 10^{2}$	${ 8}{\text{.90}} \times {10}^{ - 12}$
Square	3	2.08	0.270	0.693	${ 1}{\text{.84}} \times {10}^{0}$	0.203741
Interaction	3	18.49	2.402	6.163	${1}{\text{.64}} \times {10}^{1}$	0.000348
Residual error	10	3.77	0.490	0.377		
Lack of fit	3	3.77	0.490	1.256	${ 4}{\text{.25}} \times {10}^{29}$	$2.2 \times 10^{ - 16}$
Pure error	7	0	0	0	$\, 3.31 \times 10^{4}$	
$$\user2{Nu}/\sqrt {{\mathbf{Re}}_{\user2{x}} }$$						
Linear	3	24.6507	94.145	8.2169	$3.31 \times 10^{4}$	$2.2 \times 10^{ - 16}$
Square	3	0.036	0.137	0.012	$4.84 \times 10^{1}$	$2.94 \times 10^{ - 6}$
Interaction	3	1.492	5.698	0.4973	$2.00 \times 10^{3}$	$3.41 \times 10^{ - 14}$
Residual error	10	0.0025	0.010	0.0002		
Lack of fit	3	0.0025	0.010	0.0008	$5.44 \times 10^{27}$	
Pure error	7	0	0	0		

**Figure 10:**
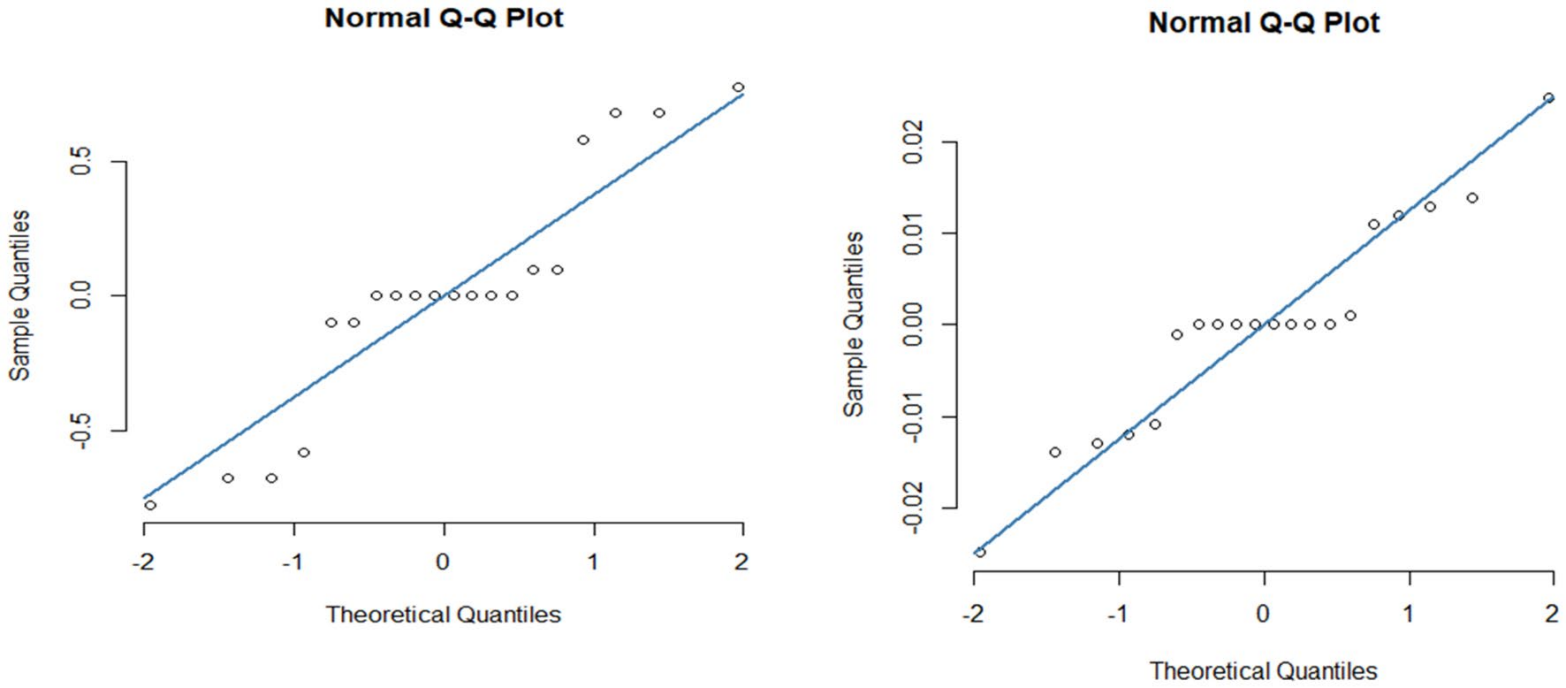
The residual normal ($Q - Q$) plot for $Cf\sqrt {{\text{Re}}_{x} }$ and $Nu/\sqrt {{\text{Re}}_{x} }.$

**Figure 11:**
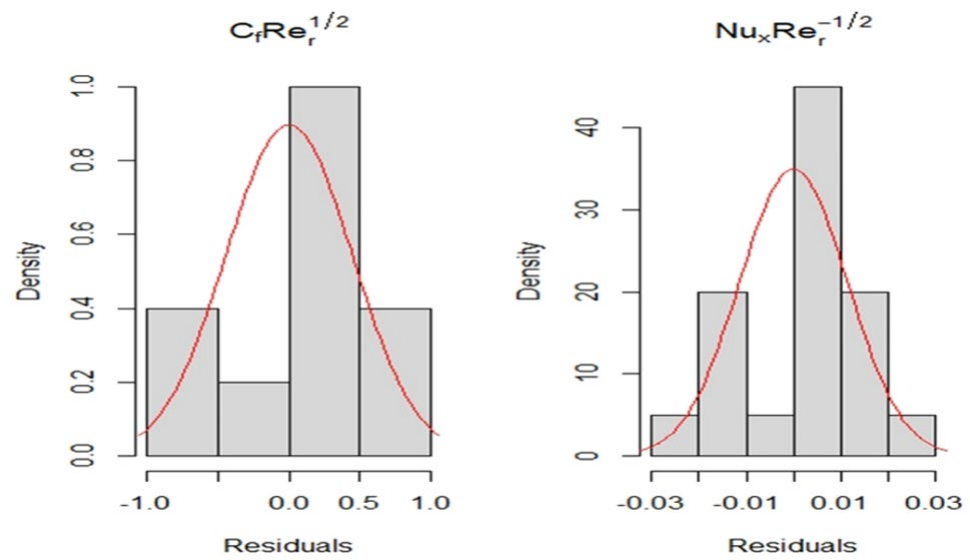
The histograms of residuals for both fitted models.

Several variables are evaluated in order to examine the quality of fit. For instance, the absence of fit has a ($p{\text{ - value}} < 0.0001$) for each of the two fitted models. Second, adjusted $R^{2}$ is used to indicate how well the models describe the deviation in response. For $Cf\sqrt {{\text{Re}}_{x} }$ and $Nu/\sqrt {{\text{Re}}_{x} }$, the adjusted $R^{2}$ is 99.5% and 99.9% respectively. As a consequence, all models account for a significant fraction of the whole range in their respective replies. Thirdly, the quality of fit is evaluated using a traditional residual $Q - Q$ plot. In an appropriate model that suitably reflects the functional connection between input parameters and response, theoretical and observed quantiles have a one-to-one correspondence. Figure 10 depicts the standard residual $Q - Q$ plot for the two models. This means that the theoretical and observed quintiles are roughly one-to-one for both models. Finally, the fitted models’ residuals are considered to be normally distributed. The straight line in the graph shows that the errors are normally distributed, which indicates that our models are properly fitted. Figure 11 depicts the residuals of the fitted models, which indicate that they all have a normal distribution. According to these assessments, both models are appropriate. The fitted models are:

$$\begin{aligned} & Cf\sqrt {{\text{Re}}_{x} } = 11.294 + 0.09677A + 4.81941B + 8.33531C + 0.02046AB \\ & \quad + 0.0756BC + 0.71662AC - 0.10258A^{2} + 0.65072B^{2} + 1.76566C^{2} \\ \end{aligned}$$

$$\begin{aligned} & Nu/\sqrt {{\text{Re}}_{x} } = 1.57933 + 0.031258A + 0.161246B + 1.74761C + 0.00786AB \\ & \quad + 0.0174BC - 0.092908AC - 0.0112A^{2} + 0.016581B^{2} + 0.556331C^{2} \\ \end{aligned}$$

A derivation of the response function is a common definition of sensitivity in terms of model variables. When compared to model vigour estimates, sensitivity analysis investigates the distinct demands offered by model output spread by input variables. In this scenario, the partial derivative of response is utilized to express the sensitivity function of the input parameters $$A\left( \phi \right),$$$$B\left( M \right),$$$${\text{and }}C\left( \gamma \right)$$, which are:

$$\begin{aligned} \frac{{\partial \left( {Cf\sqrt {{\text{Re}}_{x} } } \right)}}{\partial A} & = 0.09677 - 0.0224A + 0.02046B - 0.092908C \\ \frac{{\partial \left( {Cf\sqrt {{\text{Re}}_{x} } } \right)}}{\partial B} & = 4.81941 + 0.02046A + 1.30144B + 0.0756C \\ \frac{{\partial \left( {Cf\sqrt {{\text{Re}}_{x} } } \right)}}{\partial C} & = 8.33531 + 0.71662A + 0.0756B + 3.53132C, \\ \end{aligned}$$

$$\begin{aligned} \frac{{\partial \left( {Nu/\sqrt {{\text{Re}}_{x} } } \right)}}{\partial A} & = 0.031258 - 0.0224A + 0.00786B - 0.092908C \\ \frac{{\partial \left( {Nu/\sqrt {{\text{Re}}_{x} } } \right)}}{\partial B} & = 0.161246 + 0.00786A + 0.033162B + 0.0174C \\ \frac{{\partial \left( {Nu/\sqrt {{\text{Re}}_{x} } } \right)}}{\partial C} & = 1.74761 - 0.092901A + 0.0174B + 1.112662C. \\ \end{aligned}$$

For SFC ($Cf\sqrt {{\text{Re}}_{x} }$), the partial derivatives given above can be utilized, and relevant sensitivity measurements are derived for the LNN ($Nu/\sqrt {{\text{Re}}_{x} }$). Table 7 displays the sensitivity measurements with (B = 0) and various combinations of magnetic field parameters (A) and Biot number (C). When the sensitivity measure is positive, it suggests that the input parameters ($\phi$, $\gamma$ and $M$) improve as the response rises, and when it is negative, it implies that the input parameters ($\phi$, $\gamma$ and $M$) decrease as the response rises.

**Table 7:** Sensitivity analysis of $Cf\sqrt {{\text{Re}}_{x} }$ and $Nu/\sqrt {{\text{Re}}_{x} }$ with B = 0 and all A and C combinations.

A	C	Sensitivity					
		$\frac{{\partial \left( {Cf\sqrt {{\text{Re}}_{x} } } \right)}}{\partial A}$	$\frac{{\partial \left( {Cf\sqrt {{\text{Re}}_{x} } } \right)}}{\partial B}$	$\frac{{\partial \left( {Cf\sqrt {{\text{Re}}_{x} } } \right)}}{\partial C}$	$\frac{{\partial \left( {Nu/\sqrt {{\text{Re}}_{x} } } \right)}}{\partial A}$	$\frac{{\partial \left( {Nu/\sqrt {{\text{Re}}_{x} } } \right)}}{\partial B}$	$\frac{{\partial \left( {Nu/\sqrt {{\text{Re}}_{x} } } \right)}}{\partial C}$
−1	−1	0.169218	3.44237	4.72839	0.116306	0.110684	0.61754
	0	0.07631	3.51797	8.25971	0.02472	0.128084	1.73021
	1	−0.016598	3.59357	11.79103	−0.06951	0.145484	2.842872
0	−1	0.189678	4.74381	4.80399	0.124166	0.143846	0.634948
	0	0.09677	4.81941	8.33531	0.031258	0.161246	1.74761
	1	0.003862	4.89501	11.86663	−0.06165	0.178646	2.860272
1	−1	0.169218	6.04525	4.87959	0.132026	0.177008	0.652348
	0	0.11723	6.120285	8.41091	0.039118	0.194408	1.76501
	1	0.024322	6.19645	11.94223	−0.05379	0.211808	2.877672

**Figure 12:**
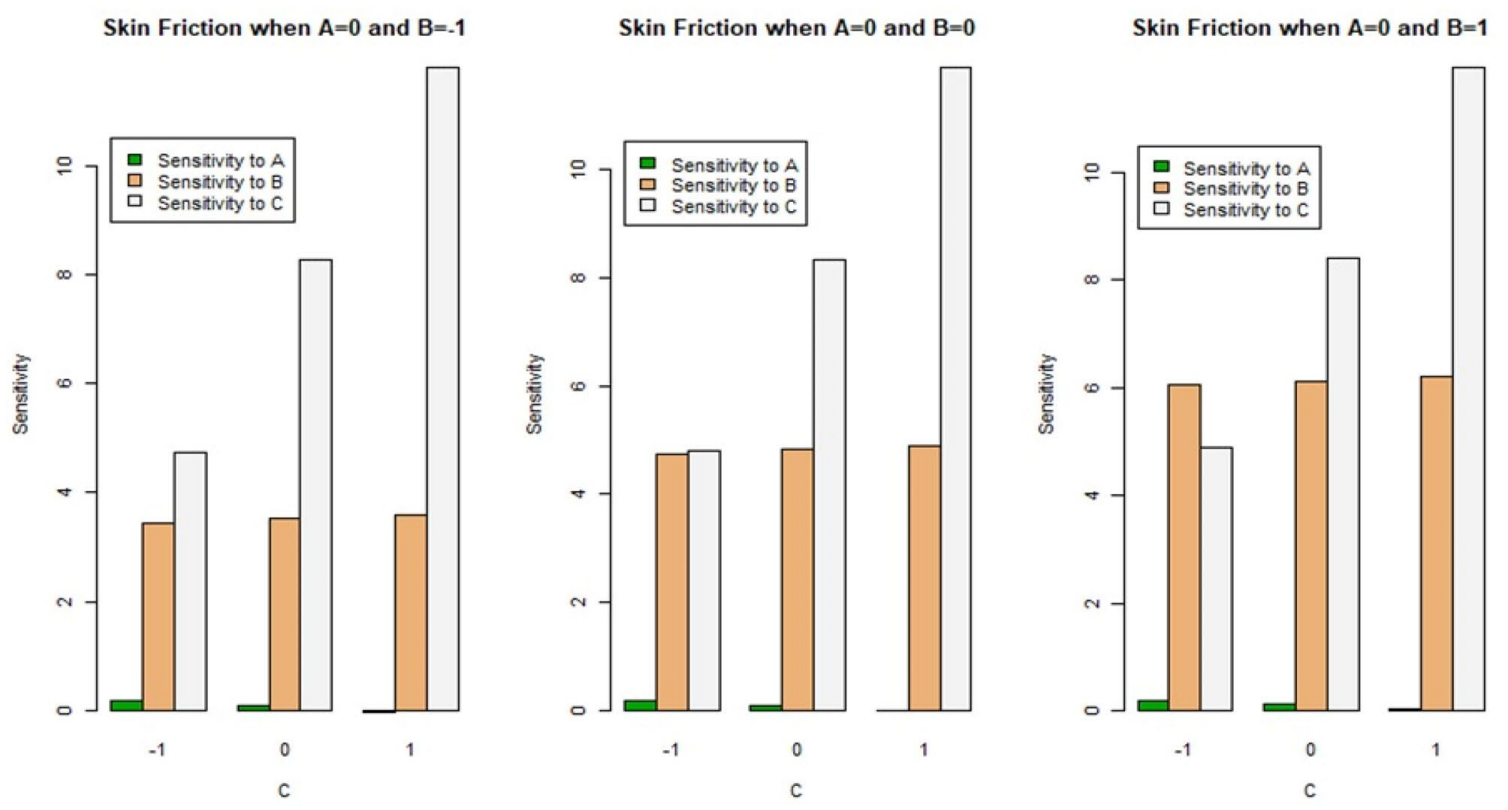
Bar chart representation of sensitivity analysis for $C_{f} \sqrt {{\text{Re}}_{x} }$ with A = 0 and all combinations of (B) and (C).

**Figure 13:**
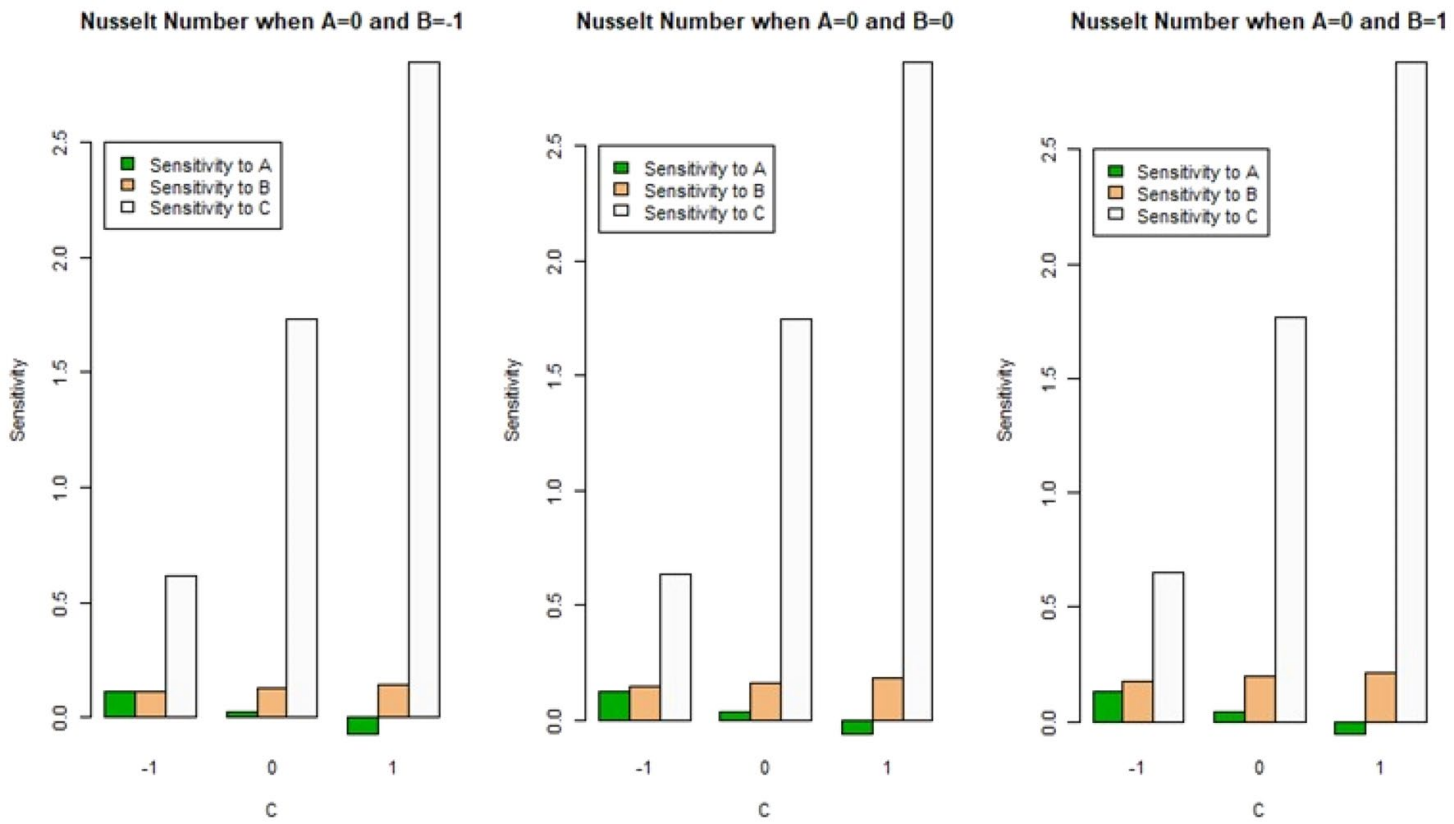
Bar chart representation of sensitivity analysis for $Nu/\sqrt {{\text{Re}}_{x} }$ with A = 0 and all combinations of (B) and (C).

Figures 12 and 13 exhibit the bar chart findings of the sensitivity analysis for better understanding. Positive sensitivity levels are depicted by upward bars in this bar plot, whereas, negative sensitivity values are represented by downward bars. Figure 12 represents the sensitivity of SFC $C_{f} \sqrt {{\text{Re}}_{x} }$ at A = 0, for different cases of (B) and (C). We observed that for all levels (low, medium, and high) of magnetic field parameter (B) and Biot number (C), there is positive sensitivity for $C_{f} \sqrt {{\text{Re}}_{x} }$. The sensitivity analysis for the LNN $Nu/\sqrt {{\text{Re}}_{x} }$ at A = 0 and all variations of magnetic field parameter (B) and Biot number (C) is shown in Fig. 13. It is noted that for all combinations of (B) with low and medium levels of (C), there is positive sensitivity for $Nu/\sqrt {{\text{Re}}_{x} }$, whereas, for all combinations of (B) with a high level of (C), there is negative sensitivity for $Nu/\sqrt {{\text{Re}}_{x} }$.

**Figure 14:**
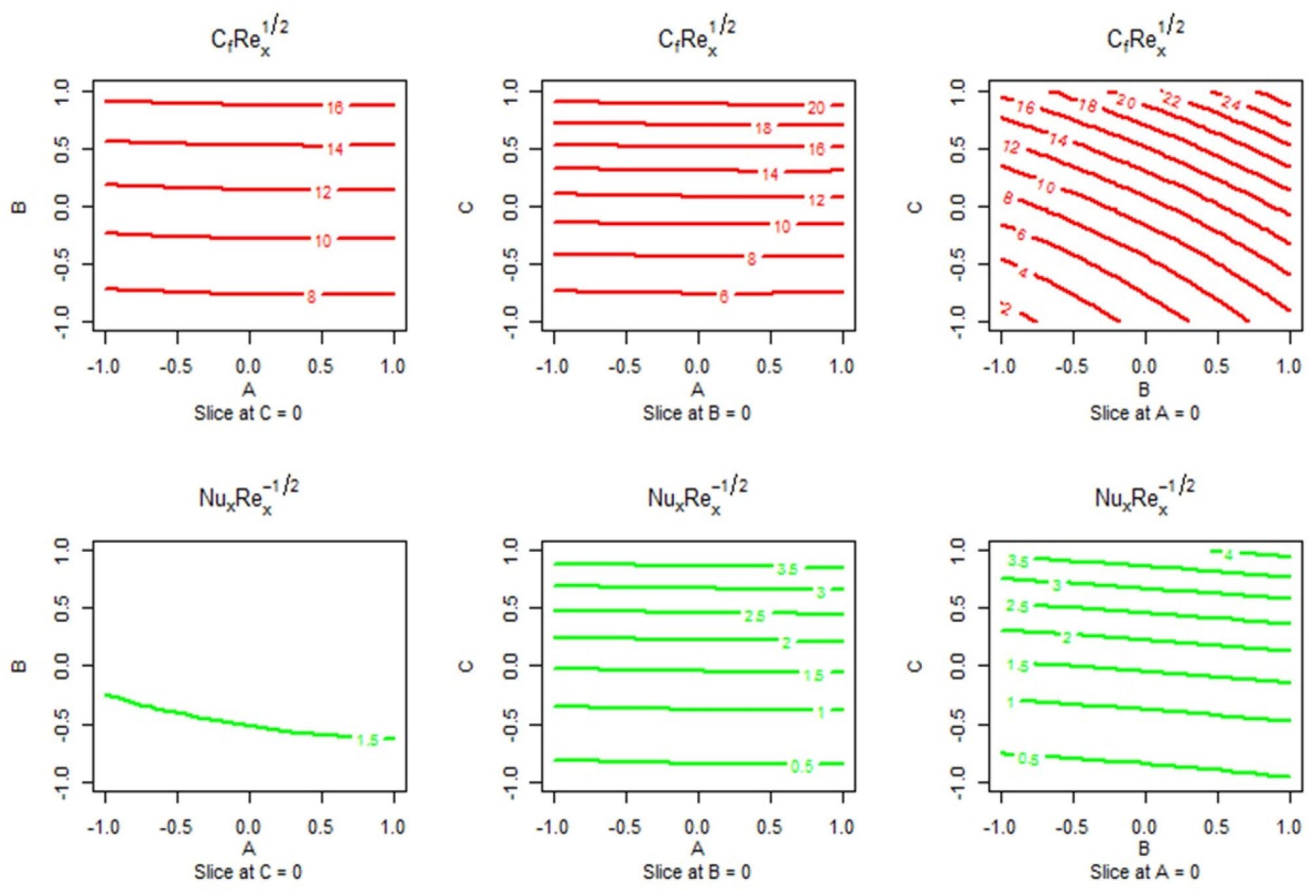
Predicted responses of $C_{f} \sqrt {{\text{Re}}_{x} }$ and $Nu/\sqrt {{\text{Re}}_{x} }$ as a function of input parameters with different combinations of (**A** & **B**), (**A** & **C**), and (**B** & **C**).

Figure 14 depicts the estimated response as a function of several parameters for input levels, for instance,$M \, \& \, \phi$,$M \, \& \, \gamma$, and $\phi \, \& \, \gamma$. The anticipated responses for SFC ($Cf\sqrt {{\text{Re}}_{x} }$) and LNN ($Nu/\sqrt {{\text{Re}}_{x} }$) are shown in Fig. 13’s top and bottom panels, respectively. When the Biot number has no impact, the SFC is maximized at the maximum level of the magnetic field parameter and nanoparticle volume fraction. The SFC is maximized at the highest level of the magnetic field parameter and Biot number when the nanoparticle volume fraction is held constant. When the magnetic field parameter remained zero, the SFC was maximized at any Biot number and maximum nanoparticle volume fraction. The lower panel depicts the anticipated response of the LNN ($Nu/\sqrt {{\text{Re}}_{x} }$). When the effects of the Biot number are maintained to a minimum, the LNN is maximize at the lowest nanoparticle volume fraction and highest magnetic field parameter values. The LNN is maximized for all Biot number and magnetic field parameter values when the effects of nanoparticle volume fraction are maintained constant. Similarly, by keeping the magnetic field parameter constant at zero, the LNN is maximized at all Biot number levels and at the maximum level of the nanoparticle volume fraction.

## Conclusions

In this work, we investigated the MHD flow of nanofluid across an exponentially extending surface with non-uniform heat flux effects. The governing equations after employing the boundary layer approximations are transformed to a system of non-dimensional equations which have been analyzed numerically, and the impacts of emerging parameters were illustrated using graphs. An experimental design and a sensitivity analysis based on Response Surface Methodology (RSM) were used to investigate the impacts of several physical factors on the skin friction coefficient and local Nusselt number, for instance, magnetic field parameter, nanoparticle volume fraction, and heat transfer Biot number. The study’s primary conclusions are as follows:

- The magnetic field parameter and the volume fraction of nanoparticles reduce fluid velocity.
- As the temperature of the fluid rises, so does the nanoparticle volume fraction and the heat transfer Biot number.
- Based on well-defined statistical measures ($Q - Q$ residual plots, hypothesis testing via *p*-value, and adjusted $R^{2}$), our models for skin friction coefficient and local Nusselt number are shown to be the best fitted models.
- The skin friction coefficient is significantly influenced by nanoparticle volume fraction, Biot number, and square of the Biot number at less than a 0.01% level of significance, while for square of the nanoparticle volume fraction and product of nanoparticle volume fraction with Biot number at a 5% level of significance, whereas, local Nusselt number is significantly influenced by magnetic field parameter, nanoparticle volume fraction, Biot number, product of nanoparticle volume fraction with Biot number, and square of Biot number, at less than a 0.01% level of significance.
- Among the significant parameters, Biot number has the highest impact on skin friction coefficient and local Nusselt number, with a coefficient of 8.3353 and 1.7477, respectively, which is also proved through sensitivity analysis.
- The sensitivity of the skin friction coefficient, as well as the local Nusselt number through Biot number, is greater than that of nanoparticle volume fraction and magnetic field parameter. For example, the results through partial derivatives for magnetic field parameter, nanoparticle volume fraction, and Biot number are (0.1692, 0.0763, 0.0166), (3.4424, 3.5180, 3.5936), and (4.7284, 8.2597, 11.7910), respectively, at the lower level of magnetic field parameter (−1) and all three levels of Biot number (−1, 0, 1). Similarly, it can be shown at other levels of magnetic field parameter as well as for local Nusselt number.
- The skin friction coefficient is positively sensitive to all combinations of all input parameters. At high levels of Biot number, the local Nusselt number displays negatively sensitive magnetic field parameters.

## Data Availability

The datasets used and/or analyzed during the current study available from the corresponding author (A. A.) on reasonable request.
